# Missing Pieces of the Puzzle to Address Market Failures for Antibiotics: Delinked Payment Systems and Insurance Value

**DOI:** 10.1007/s41669-025-00591-1

**Published:** 2025-07-02

**Authors:** Neil Hawkins, Adrian Towse, Amanda Adler

**Affiliations:** 1 Health Economics & Health Technology Assessment, School of Health and Wellbeing, University of Glasgow, Glasgow, Scotland; 2 Office of Health Economics, London, United Kingdom; 3 Diabetic Medicine and Health Policy, University of Oxford, Oxford, United Kingdom; 4 Oxford Centre for Diabetes Endocrinology and Metabolism, Churchill Hospital, Headington, Oxford, OX3 7LE United Kingdom

## Abstract

Too few companies develop new antibiotics because of the threat of market failure. To address this, a ‘delinked’ payment, distinct from the usual payment model for drugs, makes payments to manufacturers that do not depend on the volume of antibiotic prescribed. A delinked system removes incentives to overpromote antibiotic use. If total payments are high enough, this system should provide incentives for manufacturers to develop new antibiotics. This assumes that using antibiotics remains consistent with antimicrobial stewardship, a coordinated approach to prescribing antimicrobials responsibly. A delinked system can address market failure that occurs when a disproportionate degree of clinical benefit from a new antibiotic occurs after patent protection ends, reducing the ‘reward’ to the innovator. Determining value in a delinked payment system requires that the health service estimates the lifetime value of an antimicrobial product, and then decides what proportion of that value to include. These values depend in part on ‘STEDI’ values including the ‘insurance’ value new antibiotics would offer in reducing society’s risk of major health loss from possible future major episodes of antibiotic resistance. Estimating insurance value requires estimating the health consequences of catastrophic outcomes. Payments in a delinked system can incorporate an ‘insurance premium’. We use the example of the UK’s delinked payment scheme to illustrate issues and solutions.

## Key Points for Decision Makers

**Table:** 

A delinked payment model enables a company’s revenues to be brought forward into the patent period.
A process for health technology assessment including benefits at the population level in quality-adjusted life years (QALYs), above those accruing to individual patients, requires accounting for STEDI values including ‘insurance’ value.
Calculating insurance value for an antibiotic includes, but is not limited to, developing scenarios where significant antimicrobial resistance occurs and assigning probabilities and consequences in the absence of effective antibiotics; producing a ‘risk neutral’ value of a new antibiotic, and then determining the insurance value given degrees of societal risk aversion.

## There Are Too Few New Antibiotics Globally

Despite rising death rates from resistance to existing antibiotics [1, 2], too few new antibiotics are being developed, and too few companies are developing antibiotics. The causes relate to expected revenues during market exclusivity, and ‘normal’ payment arrangements. Both correlate poorly with the lifetime value of an antibiotic [3, 4].

Leonard and colleagues from the National Institute of Health and Care Excellence (NICE) note, ‘… the inadequate pipeline of new antibacterials is the result of a market entry failure where new antibacterials tend to be used as little as possible’ [5]. Antimicrobial stewardship, that is, changing prescribing practices to slow antimicrobial resistance (AMR), mandates restricting prescriptions for new antibiotics while existing ones remain effective. This limits the revenue earned when an antibiotic enters the market. As AMR worsens, both the volume of prescriptions and the added value of a novel antibiotic may increase dramatically. However, if the increases occur after a patent expires, the revenues will not be received by the innovator, but by competitors making generic versions.

Globally, 1.27 million deaths are attributable to AMR [6], and deaths are increasing in both high- and low-income countries. The need for new payment incentives for drugs to tackle AMR has been recognised repeatedly by the Group of Seven with its call for the UN to host a ‘successful high-level meeting on AMR in September 2024 that galvanizes action on this critical health, economic, and security threat’ [7]. New payment models are being explored in the USA [8], the EU [9], Canada [10], Japan [11], and Italy [12], as well as the UK model we discuss. All models involve assessing likely clinical value. As we set out below, one critical aspect of this: insurance value, requires exploring further.

## The NHS Addressed These Issues Using a Delinked Subscription Model

NICE and National Health Service (NHS) England piloted a process to evaluate and pay for antimicrobials [13] [14]. NICE and NHS England aimed, ‘… to incentivise companies to invest in researching and developing new antibiotics, helping secure much-needed alternative treatment options for NHS patients...’. They also noted, ‘… high cost and low returns associated with antibiotic research and development makes it commercially unattractive’ [15].

The pilot comprised [13]:

- A payment model with reimbursement from the NHS to the manufacturer ‘delinked’ from the volume of the antimicrobial the NHS acquires.
- A process for health technology assessment including benefits at the population level, in quality-adjusted life years (QALYs), above those accruing to the individual patients [16].

A delinked payment model enables a company’s revenues to be brought forward into the patent period of the given antimicrobial. The bespoke NICE appraisal committee was asked to determine the value of an antibiotic to the NHS over 20 years, and to assign a proportion of this value over the first 10 years, a typical period of patent exclusivity. Evidence from sales for non-antibiotic drugs informed this decision; it suggested that 59% of the value of a typical new drug to the healthcare system accrues in its first 10 years [17]. The committee chose a value of at least 60% to further the ‘pull incentive’ [18].

The delinked model is key because it discourages manufacturers from promoting the use of new antibiotics (potentially at levels above those consistent with antibiotic stewardship) with the contracts between NHS England and pharmaceutical manufacturers including agreements on stewardship, manufacturing and environmental practices, and limits on marketing [18, 19]. It also removes financial incentives on local healthcare providers to substitute cheaper generic antibiotics for more expensive newer antibiotics when the newer antibiotics are clinically indicated. Finally, it allows the insurance value of new drugs to be recognised.

If the delinked model is to encourage manufacturers to develop antimicrobials, the expected payments under the model will need to exceed the expected costs of development, accounting for the costs of capital and probability of failure at the various stages of the development process [20]. To ensure this, the UK scheme provides annual payments initially up to £10 million estimated as representing the UK’s ‘fair share’ of the typical global cost of developing a new antibiotic [21]. However, there were calls recommending that the UK pays more, noting that developing a new antibiotic may require a reward of $2 billion to $4 billion to cover costs and failed attempts [22, 23]. Revised proposals have increased the yearly maximum to £20 million [24] and increased the potential contract period to 15 years to better reflect patent life [25].

## A New Approach to Valuing Antibiotics that Incorporates ‘STEDI’ Criteria

The NICE pilot considered population-level factors specific to antibiotics: the ‘STEDI’ criteria [26]. These include:

- Spectrum value: using narrow (not broad) spectrum antibiotics to reduce AMR.
- Transmission value: reducing onward transmission of resistant pathogens.
- Enablement value: permitting treatments and medical procedures that would not be possible were antibiotics not available.
- Diversity value: increasing the range of treatment options to reduce AMR.
- Insurance value: having effective antibiotics available in case of a sudden, major or catastrophic increase in AMR.

Including STEDI values goes beyond the usual assessment of new drugs in two respects: first, instead of focusing solely on value to an individual, the approach includes benefits accruing to the population. Until now, bodies such as NICE have usually considered these effects only when looking at the population benefits from herd immunity or decreased transmission when assessing new antivirals. The second difference in the assessment relates to including societal ‘risk aversion’ to future epidemics of widespread resistant infection. Societal risk aversion could be likened to a householder’s decision to purchase fire insurance. The cost of the insurance will be greater than the average expected loss, given the probability and costs of a fire, over all households, and the administrative costs of the insurers, otherwise insurers would not offer policies. However, householders may still buy insurance, even though the expected value is less than the expected cost. In this case we would describe the householder as risk averse. Risk averse, as strictly defined in the field of health economics, is equivalent to a concave utility function [27].

Likewise, a government might decide to invest more than the expected ‘cost’ of an AMR outbreak, based on its probability and consequences, into measures to mitigate the risk of an outbreak of AMR given the severity of its consequences.

## STEDI Criteria in the NICE Pilot

Considering STEDI criteria should increase the public’s confidence that the estimated benefits to the population are valid. NICE and NHS England can use these estimates, derived using net-monetary benefits, to set a maximum acceptable payment for a product. Payments to manufacturers higher than this would reduce health by displacing investment elsewhere. Including STEDI criteria in calculating the maximum acceptable payment is associated with an opportunity cost; however, only one of the criteria, insurance value, does not increase average expected health gain, but would increase health in the event of a major outbreak of AMR as we describe below. In short, insurance value adds to the payment, but not the expected health gain.

In estimating STEDI values, the NICE committee recognised that the values depend on the extent that clinicians will prescribe the new antibacterial, its characteristics, and the nature and growth of infections resistant to current treatments. The NICE/NHS England ‘lessons learned’ report [28] noted that STEDI values were, ‘… the most prominent aspects of value not fully captured by modellers…’, which supported NICE’s decision to convene a special committee to take a view on their value.

## Insurance and the Degree of Risk Aversion the NHS Should Adopt

The ‘lessons learned’ report [28] identified, ‘… enablement and insurance value as the most important drivers of value’.

Insurance value is distinct from other STEDI criteria because it does not depend on the volume of antibiotic used; it is also the most difficult to value as it requires estimating the probability and consequences of AMR outbreaks. It relates to the fundamental reason why people worry about too few new antibiotics, namely, the concern about future catastrophic increases in AMR. Estimating insurance value requires predicting a range of future trends in AMR, including extreme scenarios. It is difficult to assess the magnitude of society’s risk aversion and the appropriate ‘premium’ to pay for a new antibiotic over its expected value. Tackling low probability scenarios for outbreaks of AMR-related infections that cause irreversible or catastrophic losses requires investing in new antibiotics to ‘buy’ an expectation (insurance) that antibiotics in severe outbreak scenarios will improve outcomes. This is neither undertaken nor required for drugs in most other disease areas. A parallel would be the valuation of measures undertaken to mitigate the risks associated with possible future pandemics.

Calculating insurance value to inform NHS payments includes estimating the ‘risk aversion’ the health system should adopt. It is unlikely that society will risk future illness and death from catastrophic increases in AMR, even if the likelihood is low. Risk-aversion means that society ascribes a ‘total’ value of a new antibiotic greater than the (modelled) ‘expected’ value, estimated by multiplying the value of an antibiotic in reducing drug-resistant-associated illness and death by the probability of each of these occurring [29]. This difference between total and expected value reflects ‘insurance value’. If policy makers consider only expected value, they assume that society is risk neutral. A risk neutral individual would pay no more that £100 to avoid a 1 in 10,000 chance of losing £1 million. A risk neutral individual would not purchase insurance, as the insurance premium will be at least as large as the expected value of the pay-out. Therefore, when including administrative costs charged by the insurer, a risk neutral individual will never be better off with insurance than without. Risk neutrality is the usual stance adopted by public policy makers, including NICE. In extreme circumstances, society will expect governments to invest in strategies to prevent and mitigate damage. For AMR, the state may reasonably take a precautionary, risk averse approach when investing in new antibiotics, and ‘… pay more, and possibly much more, than decision theory would allow’ [30].

In this regard, antibiotics and infection control bear a striking resemblance to the firefighting infrastructure: the microbiology laboratory serves as the smoke detector, medical personnel are the fire fighters, and antibiotics supply the water [20]. All these elements must be established before the fire (the infection) starts. This is because buildings burn (and patients die) more quickly than an infrastructure can be built or strengthened. For example, an outbreak of plague in India in 1994 caused many deaths [31] and devastating costs to the local economy [20]. The availability of an effective therapy would doubtless have reduced the overall level of public anxiety; however, the insurance value of the drug would have been present whether or not the actual epidemic had occurred, just as the fire department is needed even if no fires occur in a community. Calculations of the insurance value of antibiotics are in their preliminary stages [32], but the consequences of not having insurance should encourage participation in a worldwide scheme ensuring antibiotic availability.

The UK Government has analysed similar scenarios using a risk matrix [33]. There is, however, a likelihood of global spread. An analogy is the need to be prepared to fight a war on multiple fronts.

Calculating insurance value for an antibiotic entails:

- Developing scenarios where significant AMR occurs and assigning probabilities and consequences in the absence of effective antibiotics.
- Producing a ‘risk neutral’ value of a new antibiotic.
- Estimating the ‘insurance premiums’ relevant for different degrees of risk aversion to the consequences of the scenarios developed.
- Agreeing what degree of risk aversion is appropriate for society and affordable to payers.

The question is, are payers willing and able to pay for the full insurance value?

For the antibiotic ceftazidime-avibactam (CAZ-AVI), the expected value of the new antibiotic estimates the consequences of emerging resistance when treating complicated urinary tract infections in the absence of the availability of the new antibiotic. When the NICE committee was presented with scenarios where the probability of a major AMR event occurring in the 10 year horizon was assumed to be 1%, the value of the new antibiotic was estimated as high as 5839 QALYs, reflecting the benefits it would deliver in this scenario—if it occurred—as compared with an expected value of 58 QALYs [18]. By ‘expected value’, we refer (as stated earlier) to the average value over all of the uncertain population outcomes, taking into account their estimated likelihood of occurring. Of note, the expected value of 58 QALYs, and the conditional (if the event occurs) value of 5839 QALYS already includes four ‘STEDI’ values (spectrum, transmission, enablement and diversity) plus the ‘usual’ individual health gains. If society were to seek to mitigate this event, which has a 1% chance from occurring, it would be willing to pay more than the expected value to encourage investment in new antibiotics. The maximum insurance value of CAZ-AVI would reflect the incremental health gain of 5839 QALYs minus 58 QALYs = 5781 QALYs; with a value of £20,000 per QALY this equates to £115.6 million. We do not suggest that the risk aversion associated with the total mitigation of an event with a probability of 1% is the correct degree of risk aversion, or that it will be necessary to spend £115.6 million to ‘insure’ against the event, we simply illustrate the potential size of the effect of taking insurance value into account to mitigate an adverse event with an estimated 1% chance of occurring. Note that the event probability does not directly affect the value of the insurance, but it might influence whether society is willing to pay the insurance ‘premium’. This will depend on society’s agreed degree of risk aversion, and the loss associated with the event, equal to the maximum one would pay for insurance.

Identifying a plausible degree of risk aversity to estimate insurance value should draw on the approach used by the government in assessing major risks. Of note, the Integrated Review Refresh, which updates the government’s defence, development and foreign policy priorities, does not include AMR explicitly, but, instead ‘… significant biological risk, no matter how these occur and no matter who and what they affect’ [34]. So, it is not currently clear how the government will evaluate risks related to AMR.

We also recognise there are scenarios that accelerate AMR, dubbed ‘black swan’ events, where new resistance genes escape from environmental bacteria and are picked up by ‘… promiscuous mobile elements and epidemic strains’ [35]. Yet, these scenarios cannot be dealt with via a delinked subscription incentive alone when a new antibiotic may not be effective against these never-before-seen events. Agencies dealing with global health are addressing this, including the Coalition for Epidemic Preparedness Innovations (CEPI); the Biomedical Advanced Research and Development Authority (BARDA) within the US Department of Health and Human Services (US HHS); and the Health Emergency Response Authority (HERA), a European Commission initiative.

## Ways Forward

NICE and NHS England have developed a ‘more pragmatic approach’, a points-scoring system in which the Antimicrobial Evaluation Panel assesses antibiotics including STEDI elements [36, 37]. The new score depends on: relative effectiveness and unmet clinical need, pharmacological benefit and health system benefit. The score places antibiotics into one of the four reward bands reflecting NHS payment to manufacturers of between £5 million and £20 million per year [24]. The subscription contract is for an initial 3-year period extendable to 15 years to cover the period of patent exclusivity [36]. This model replaces the QALY-based health technology assessment used in the pilot.

NICE and NHS England developed its scoring using experts and a ‘swing-weighting’ approach to multi-criteria decision making (MCDA) grounded in decision theory. A MCDA process can perform well, but, it is not grounded in the NICE’s preferred currency of value, the QALY, used to assess other health interventions; instead, the scoring system uses an anchor reference of annual NHS payments to manufacturers. It is not clear what would happen if the UK’s contribution to global antibiotic development needed to increase, particularly if the insurance value of a new antibiotic were high. Indeed, it is not clear to us how well the scoring system can reflect insurance value.

We support this progress. We also conclude that using a scoring system should be time limited. We anticipate that the process will evolve, informed by research, beyond a points-based system to one which uses QALY-based assessment. There is a particular need for research on estimating insurance value, the relevant degree of risk aversion and the degree of uncertainty related to the rate of growth of AMR. Converting all STEDI benefits, including insurance value, into the common currency of QALYs will then mean that, like other health interventions entering the NHS, assessing new antimicrobials can be anchored in the threshold used by NICE, while also encouraging innovators to develop much needed and eagerly awaited new antibiotics.

## Declarations

### Availability of Data and Material

Materials are publicly available as per references.
